# Use of Poloxamer 188 Dressing in the Treatment of Facial and Scalp Burns in Pediatric Patients: A Case Series

**DOI:** 10.7759/cureus.103551

**Published:** 2026-02-13

**Authors:** Juan Manuel Ascencio Reyes, Judit Alejandra Esparza Estrada, Juan Eduardo González Aboytes, Miguel Eduardo Hernández Inzunza, Adolfo Ruiz Gutiérrez, Jesús Nezahualcóyotl Briseño Villanueva, José David Medina Preciado, Mercedes Vanessa Navarro Muñoz, Rodolfo Ariel Miranda Altamirano

**Affiliations:** 1 Centro Universitario de Ciencias de la Salud (CUCS), Universidad de Guadalajara, Guadalajara, MEX; 2 Centro Universitario de Tonalá (CUT), Universidad de Guadalajara, Tonalá, MEX; 3 Unidad de Atención Integral a Niños, Niñas y Adolescentes con Quemaduras, Antiguo Hospital Civil de Guadalajara "Fray Antonio Alcalde", Guadalajara, MEX

**Keywords:** burns, dressings, facial burns, hydrogel dressing, pediatric burns, poloxamer, wounds

## Abstract

Objective: To report the use of dressings with a surfactant component in the treatment of facial and scalp burns in pediatric patients and their progression in the epithelization process.

Methodology: Retrospective observational study analyzing patients treated in our unit from September to December 2024. Pediatric patients with superficial and deep second-degree facial burns were included, and a Poloxamer 188-based dressing (PluroGel©; Medline Industries, Northfield, IL, US) was applied until complete epithelialization of the lesions.

Results: Seven pediatric patients with second-degree facial and/or scalp burns were treated with a Poloxamer 188-based dressing. The mean epithelialization time was 11.9 ± 3.3 days. Superficial burns healed faster (9.5 ± 4.9 days) than mixed-depth burns (12.8 ± 2.6 days). No adverse reactions or infections were observed. Verbal patients described the application as pleasant and reported reduced pruritus.

Conclusions: The use of a Poloxamer 188-based dressing in pediatric facial and scalp burns was well tolerated and associated with favorable healing in anatomically sensitive areas. Its thermoresponsive micellar properties support atraumatic wound management and timely epithelialization.

## Introduction

Burns are injuries to the skin or other tissues resulting from energy transfer, which cause tissue damage and destruction. These injuries can result from friction, extreme cold, heat, radiation, chemicals, or electricity. Nonetheless, the most frequent cause of burns is exposure to heat from hot liquids, solid objects, or flames [1].

Burns are the fifth most common cause of non-fatal childhood injuries. Moreover, burn-related trauma is the sixth most common type of pediatric trauma and is responsible for 5% of injury-related fatalities in children and adolescents aged 0 to 19. Globally, burns represent a significant public health challenge, resulting in approximately 180,000 deaths each year [2,3].

Head burns, particularly those involving the face and scalp, are especially critical due to the potential for long-term aesthetic and functional impairments. Despite burns in these regions, the face and scalp have distinctive specific anatomical features that support protection and recovery. The extensive vascularization of these areas allows for initial heat dissipation, limiting thermal damage and promoting cellular regeneration. In particular, the scalp, with its high density of hair follicles and sebaceous glands, plays a crucial role in facilitating skin re-epithelialization. While conservative management is often preferred, the application of dressings can accelerate wound healing, shorten recovery time, and decrease the risk of infection [4,5].

Poloxamer 188 is a non-ionic surfactant used as a primary component in wound and burn dressings. The product PluroGel© (Medline Industries, Northfield, IL, US) is a non-ionic surfactant hydrogel dressing containing Poloxamer 188, characterized by a micellar matrix and inverse thermosensitivity. The surfactant molecules form non-covalent cross-links to create the micellar matrix, where the hydrophilic surface of the micelles facilitates interaction with the wound, while the hydrophobic core of the micelles absorbs exudate and necrotic debris. The inverse thermosensitivity of the product provides the unique feature of maintaining a low-viscosity state at room temperature, while transitioning to a more liquid state at lower temperatures. As a hydrogel, it maintains a moist wound environment and allows for atraumatic dressing changes when saline solution is applied. Regarding its use in burns, the product is suitable for application in first-degree to deep second-degree burns [6,7]. 

## Materials and methods

This article is presented as a retrospective observational study analyzing patients treated in our unit (Unidad de Atención a Niñas, Niños y Adolescentes con Quemaduras, Hospital Civil de Guadalajara, Fray Antonio Alcalde) from September to December 2024.

Subject selection

We included patients under 18 years of both sexes treated between September and December 2024. The selected patients had superficial and deep second-degree burns in the facial and scalp region, regardless of burn etiology. We excluded patients with first- and third-degree burns, as well as those who received a different type of dressing on the facial or scalp area during treatment.

Assessment of burn surface area and depth

The assessment of burn depth was based on the Converse-Smith scale [8]. The total body surface area affected by the burn was determined using the Wallace rule (to patients aged ≥15 years ) and the Lund and Browder charts (to patients <15 years) [9], which were applied as established clinical calculation methods without reproducing copyrighted figures or diagrams. Additionally, the E-Burn Premium© application (BreakFirst-Hôpital Saint Joseph Saint Luc, Lyon, France) [10], a copyrighted software legally purchased by the authors, was used exclusively as a complementary clinical support tool to assist in burn surface area estimation. No images, tables, screenshots, or proprietary content from the application were reproduced or included in this manuscript.

Dressing application 

Following initial patient management and stabilization upon hospital admission, early burn wound excision was performed once the patient was hemodynamically stabilized, within the first 24-72 hours, under general anesthesia. The first application of the dressing occurred in the operating room immediately after the procedure. A 3-5 mm layer of the Poloxamer 188-based dressing (PluroGel©), pre-cooled to a temperature of 2-3°C, was applied. Subsequent applications were conducted in the patient’s room without the need for anesthesia, relying solely on analgesia. The gel was reapplied every 24 hours using sterile gloves. All subsequent applications followed the same protocol, whether performed in the operating room or the patient’s room, and were applied directly to the lesions. In cases of excessive exudate, the affected area was cleansed with distilled water before reapplication of the dressing. The dressing regimen continued until complete epithelialization of the lesions was achieved.

Statistical analysis

Given the retrospective, observational design of this case series and the limited sample size, a descriptive statistical approach was employed. Continuous variables, including age and time to complete epithelialization, were summarized using measures of central tendency and dispersion, specifically mean, standard deviation, and range. Categorical variables such as sex, burn depth, burn etiology, and affected anatomical regions were described using absolute frequencies and percentages. The time to epithelialization was descriptively analyzed both globally and after stratification according to burn depth (superficial versus mixed superficial and deep second-degree burns) and burn etiology, in order to explore healing patterns within the cohort. No inferential statistical analyses were performed. All data were systematically recorded and organized, and the results are presented in a summary table (Table 1).

**Table 1:** Summary of Pediatric Facial and Scalp Burn Cases Treated With a Poloxamer 188 Hydrogel Dressing S = Superficial second-degree; D = Deep second-degree; %TBSA = Percentage of Total Body Surface Area.

Case	Sex	Age (years)	%TBSA	Facial burn depth (s/d)	Etiology	Affected region(s)	Epithelialization time (days)
1	F	4	19	S	Oil burn	Face	6
2	M	1	30	S + D	Scald burn	Face, scalp	14
3	F	6	30	S	Scald burn	Face, frontal scalp	13
4	M	16	71	S + D	Direct flame burn	Face	10
5	M	5	3	S + D	Direct flame burn	Face, eyelid	10
6	M	7	23	S + D	Direct flame burn	Face, scalp	15
7	F	16	26	S + D	Direct flame burn	Face, scalp	15

Image documentation

The photographs were taken with a Canon EOS Rebel T6 camera (Tokyo, Japan) in automatic mode. All images used were taken with prior written informed consent and authorization from the parents of the minors.

## Results

Patients were evaluated during the period from September to December 2024. The mean age of the cohort was 7.9 years (range, 1-16 years). Of the total cases, four (57.1%) patients were male and three (42.9%) were female. Regarding burn etiology, one (14.3%) patient sustained a burn caused by contact with a high-density liquid (oil), two (28.6%) patients presented scald burns, and four (57.1%) patients had burns secondary to direct flame exposure.

None of the patients had previously diagnosed comorbidities or reported the use of medications before injury. During hospitalization, no adverse reactions related to the application of the Poloxamer 188-based dressing were documented. Additionally, no clinical evidence of infection was observed in any of the treated facial or scalp areas throughout the course of treatment.

The mean time to complete epithelialization of facial and scalp lesions was 11.9 ± 3.3 days (range, 6-15 days). When stratified according to burn depth, patients with exclusively superficial second-degree burns (2/7; 28.6%) demonstrated a shorter mean epithelialization time of 9.5 ± 4.9 days, whereas patients with mixed superficial and deep second-degree burns (5/7; 71.4%) exhibited a longer mean epithelialization time of 12.8 ± 2.6 days.

Further stratification by burn etiology revealed differences in healing patterns. The patient with a high-density liquid (oil) burn (1/7; 14.3%) achieved complete epithelialization within six days. Patients with scald burns (2/7; 28.6%) had a mean epithelialization time of 13.5 ± 0.7 days, while those with direct flame injuries (4/7; 57.1%) showed a mean epithelialization time of 12.5 ± 2.9 days, with greater variability observed within this group.

**Figure 1:**
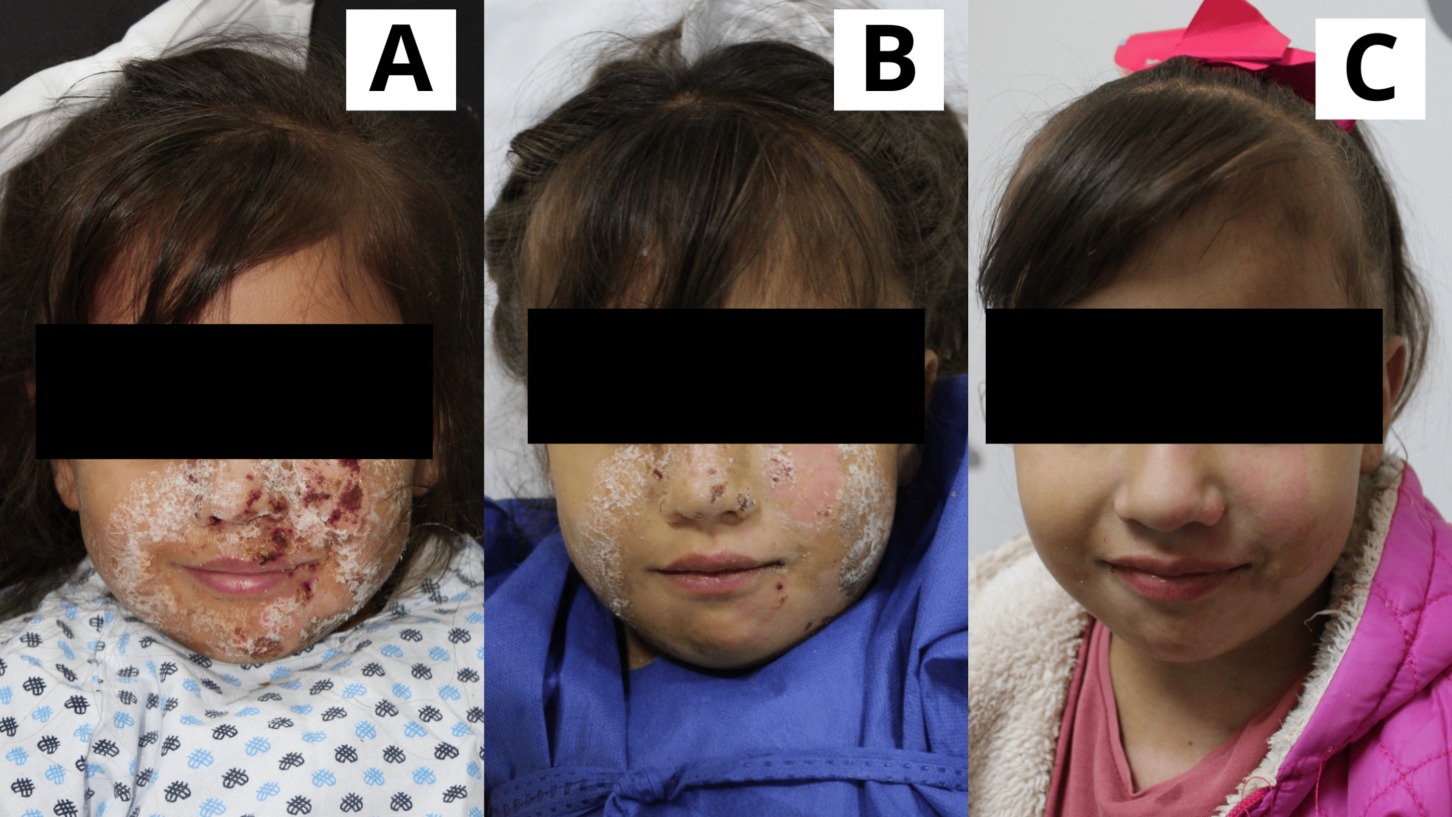
Case 1 Four-year-old female patient with a diagnosis of superficial second-degree burn caused by high-density liquid, affecting the facial region. A: Day 1: Early burn excision was performed. Subsequently, the first application of the Poloxamer 188-based dressing was carried out; whitish residues characteristic of the gel were observed. B: Day 3: The lesions appeared almost completely epithelialized, with the presence of some scabs and residual whitish material from the gel application. C: Day 6: The patient attended a follow-up consultation. Lesions were fully epithelialized.

**Figure 2:**
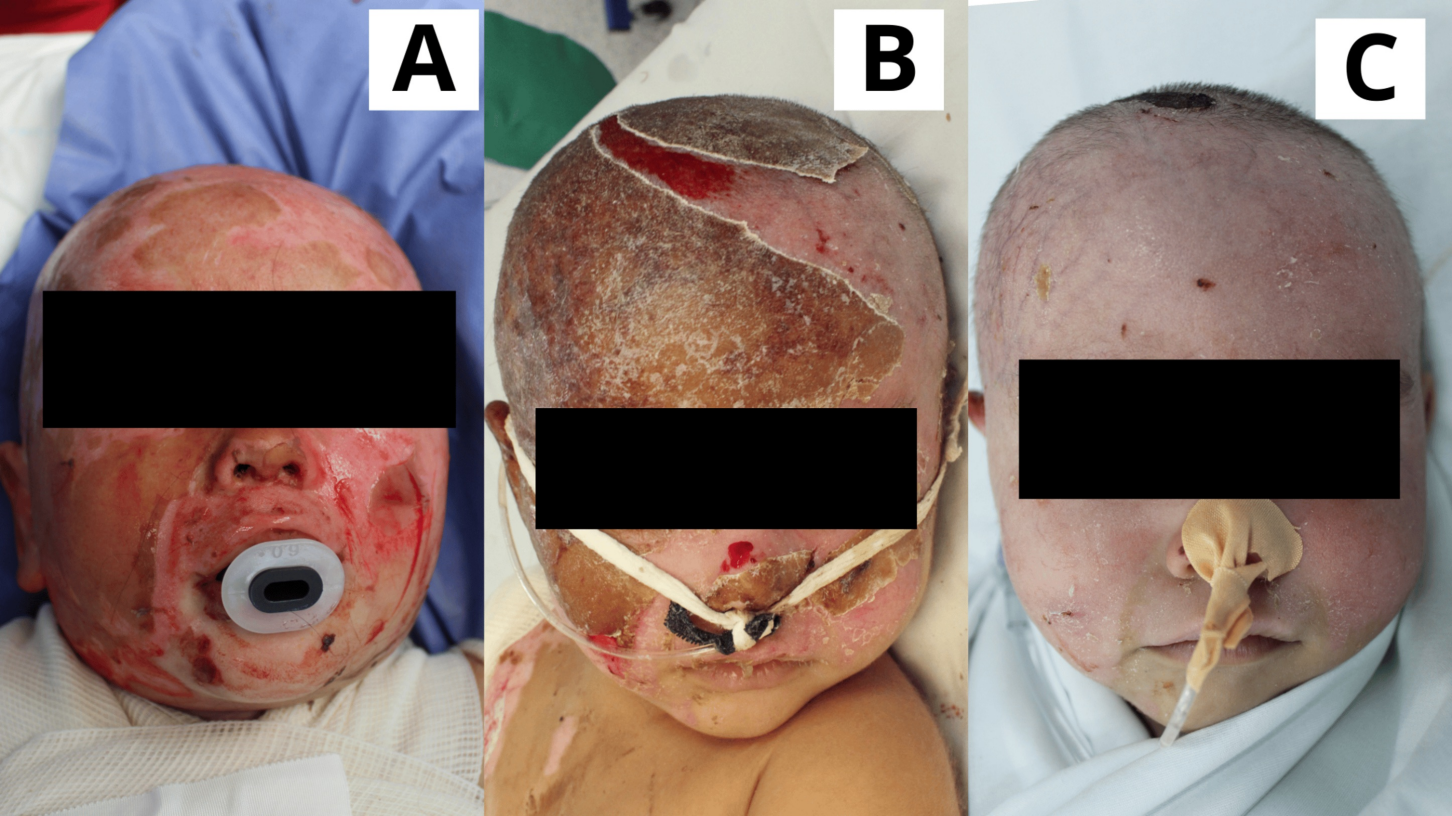
Case 2 One-year-old male patient with a diagnosis of superficial and deep second-degree scald burn affecting the facial region and scalp (frontal, parietal, and temporal areas). A: Day 1: Scalp hair was trimmed, followed by early burn excision. The product was applied. A superficial second-degree burn was observed in the facial and scalp areas, with a localized area of deep second-degree burn. B: Day 5: Lesions appeared epithelialized or in the process of epithelialization. Solidified gel was present; beneath it, wounds were progressing through the epithelialization phase. A raw area on the scalp, corresponding to the deep second-degree burn, remained visible. C: Day 10: Facial lesions were fully epithelialized. Scalp lesions were nearly completely epithelialized, with a scab present over the site of the deep second-degree burn; epithelialization was ongoing beneath the scab.

**Figure 3:**
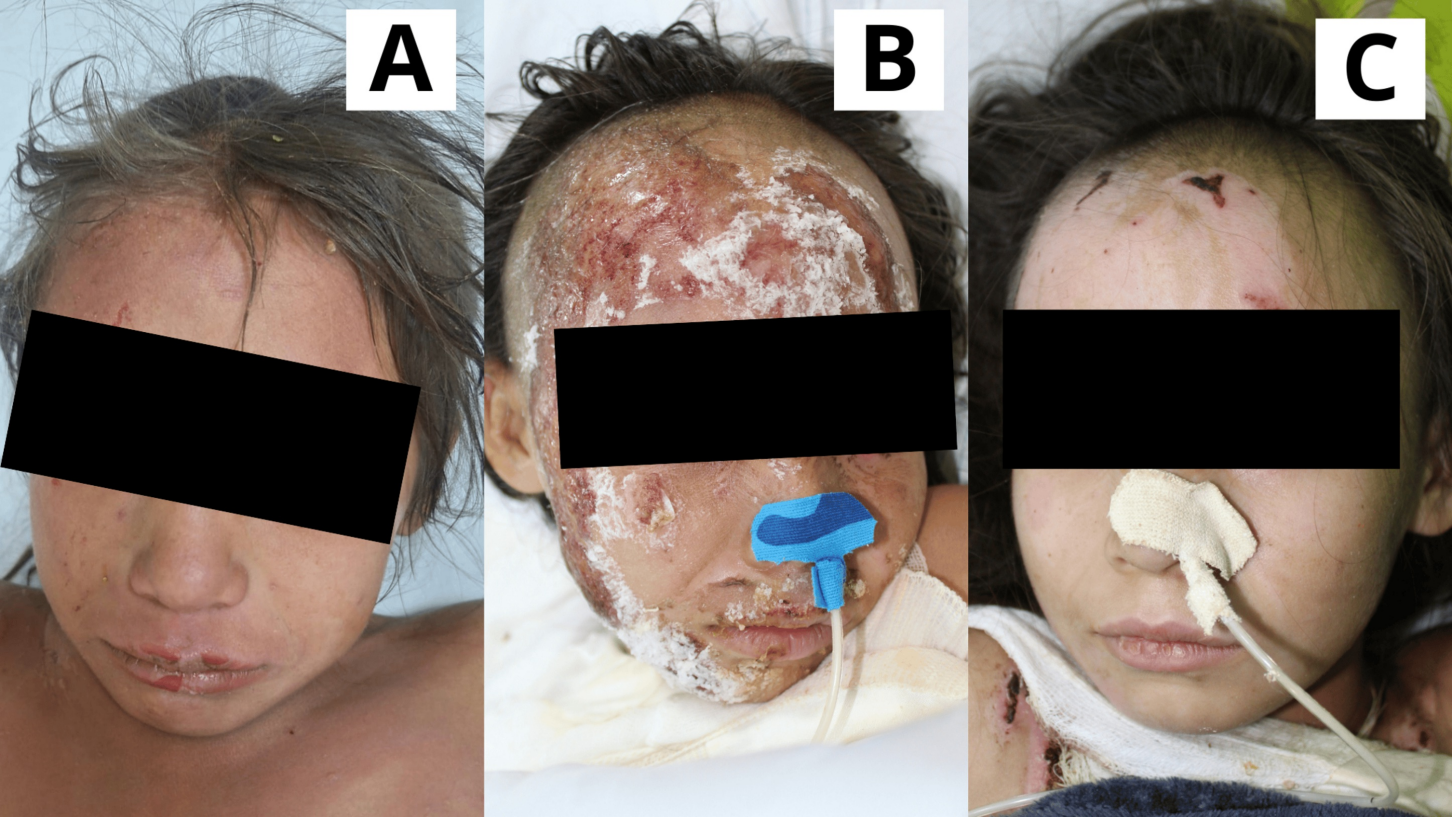
Case 3 Six-year-old female patient with a diagnosis of scald burn affecting the facial region and frontal scalp. A: Day 1: Scalp hair was trimmed, followed by early burn excision of the lesions. The product was applied. A superficial second-degree burn was observed in the facial area and frontal scalp. B: Day 6: Lesions showed the presence of scab and solidified gel; beneath the gel, wounds were progressing through the epithelialization phase. C: Day 13: Facial and scalp lesions were fully epithelialized.

**Figure 4:**
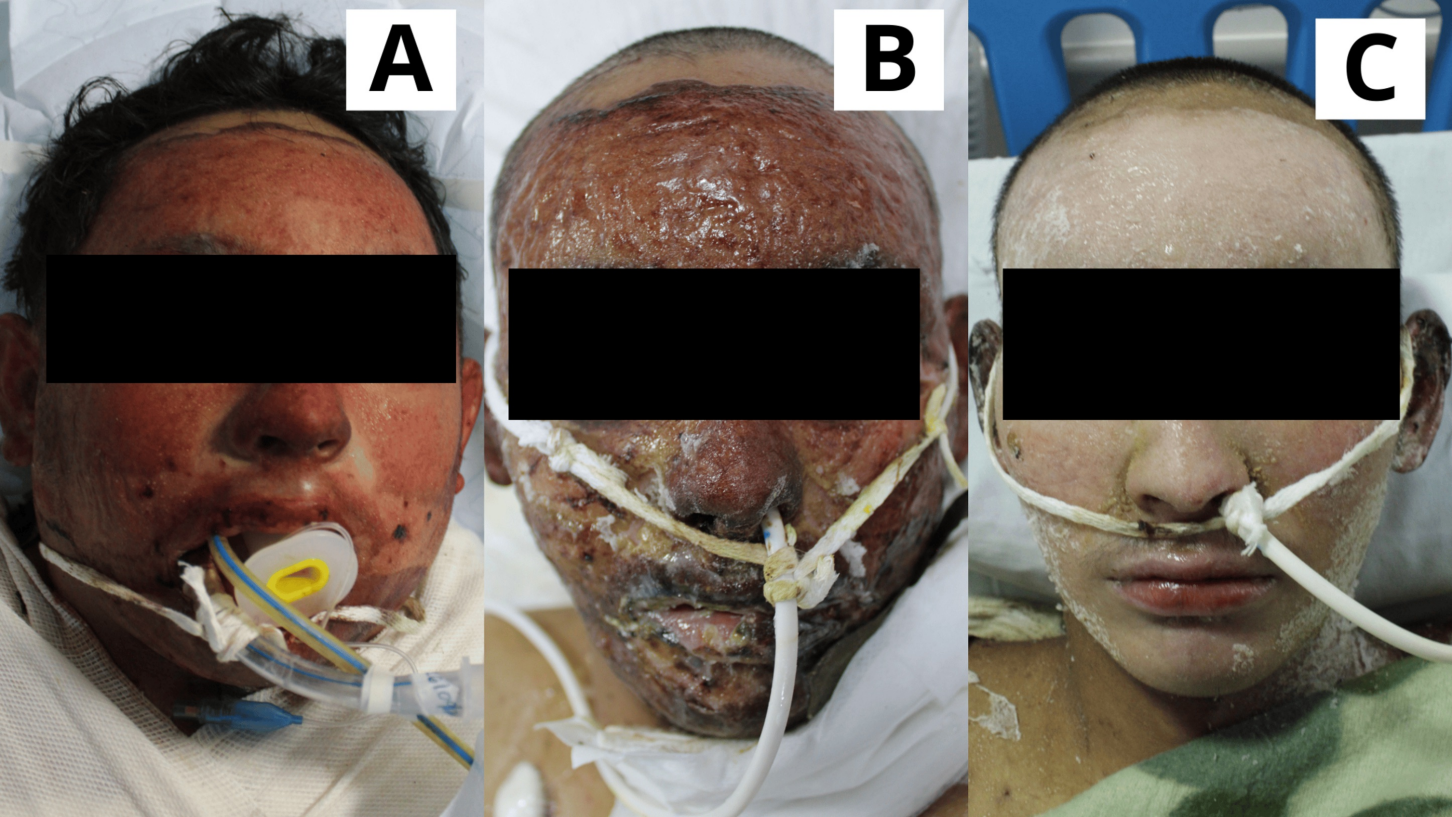
Case 4 Sixteen-year-old male patient with a diagnosis of facial burn caused by a Molotov cocktail, presenting with superficial and deep second-degree lesions. A: Day 1: Scalp hair was trimmed, followed by early burn excision. The product was applied. Both superficial and deep second-degree burns were observed in the facial area. B: Day 5: Lesions showed the presence of scab and solidified gel; beneath the gel, wounds were progressing through the epithelialization phase. Epithelialization was evaluated progressively as the gel underwent its expected gradual detachment. C: Day 10: Facial and scalp lesions were fully epithelialized.

**Figure 5:**
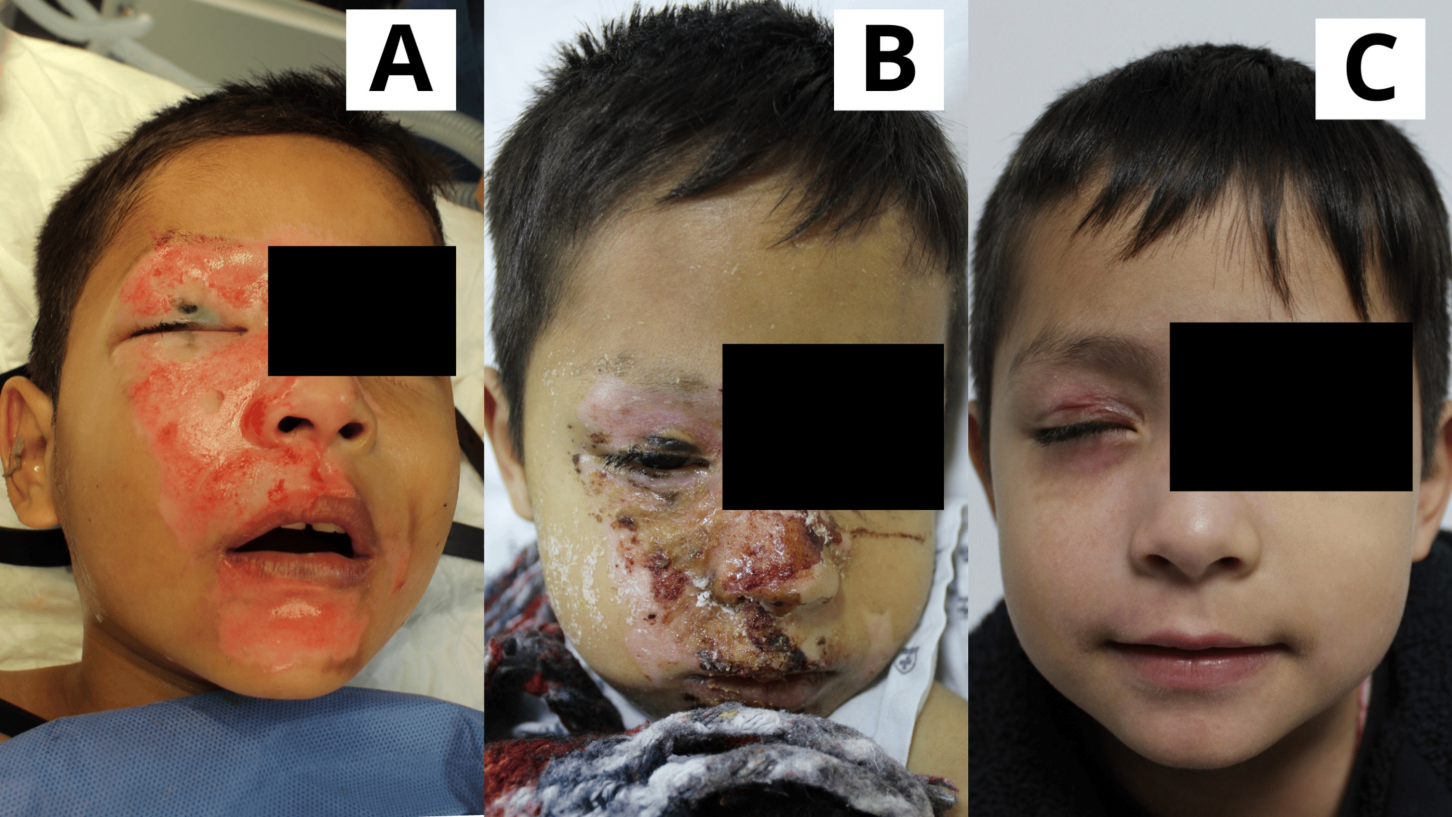
Case 5 Five-year-old male patient with a diagnosis of facial burn caused by direct flame, presenting with superficial and deep second-degree burns, including involvement of the right eyelid. A: Day 1: Early burn excision was performed, and the Poloxamer 188 dressing was applied, revealing superficial and deep second-degree burns. Notably, the burn involved the right eyelid. B: Day 5: Lesions were progressing through the epithelialization phase, with remnants of the dressing still present. C: Day 10: Facial lesions were fully epithelialized.

**Figure 6:**
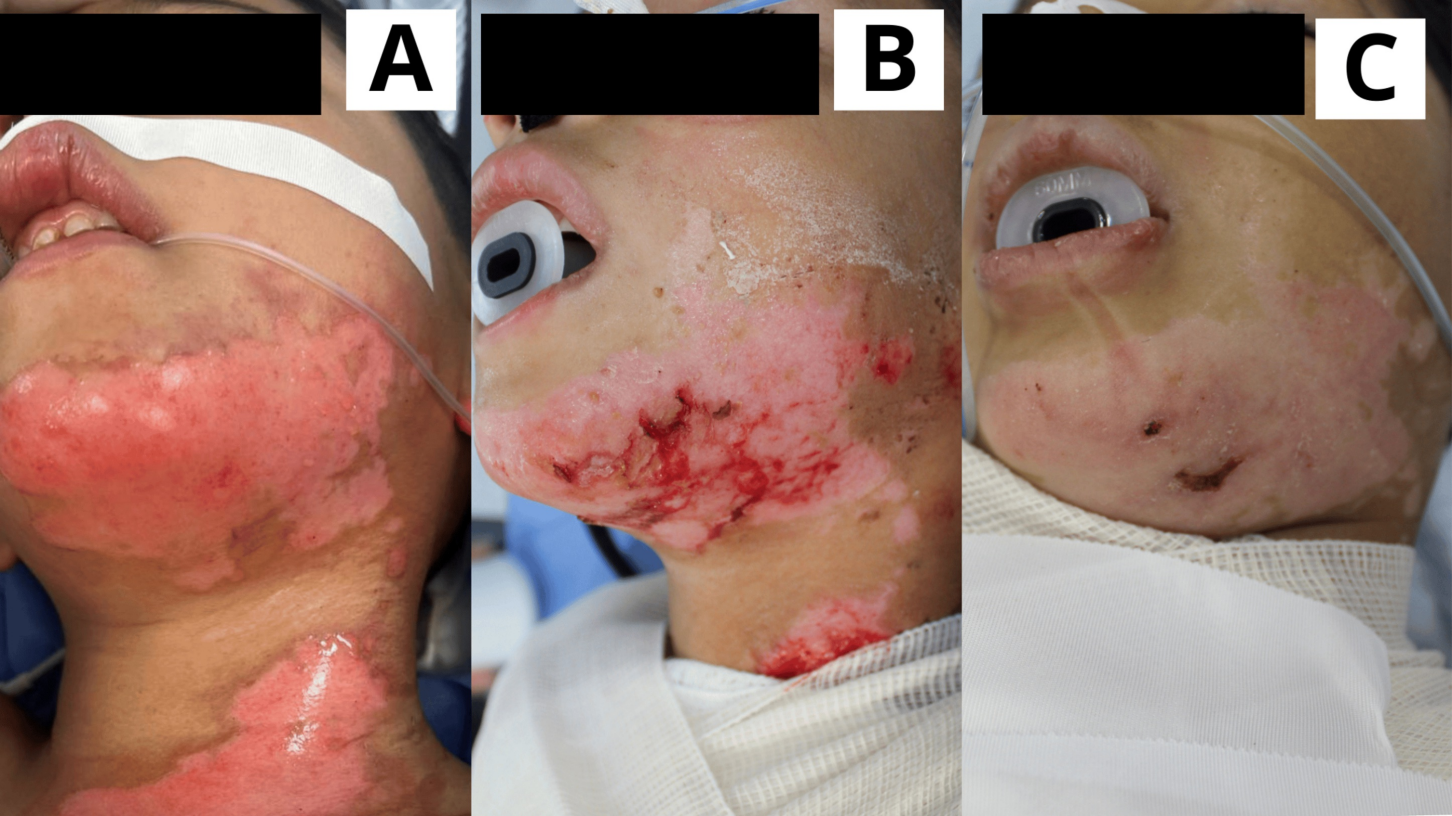
Case 6 Seven-year-old male patient with a diagnosis of a direct flame burn involving the left mandibular area, classified as superficial and deep second-degree. A: Day 1: Early burn excision was performed, and the Poloxamer 188 dressing was applied. B: Day 5: The lesion showed areas of epithelialization alongside remaining raw areas. C: Day 15: The lesion was completely epithelialized.

**Figure 7:**
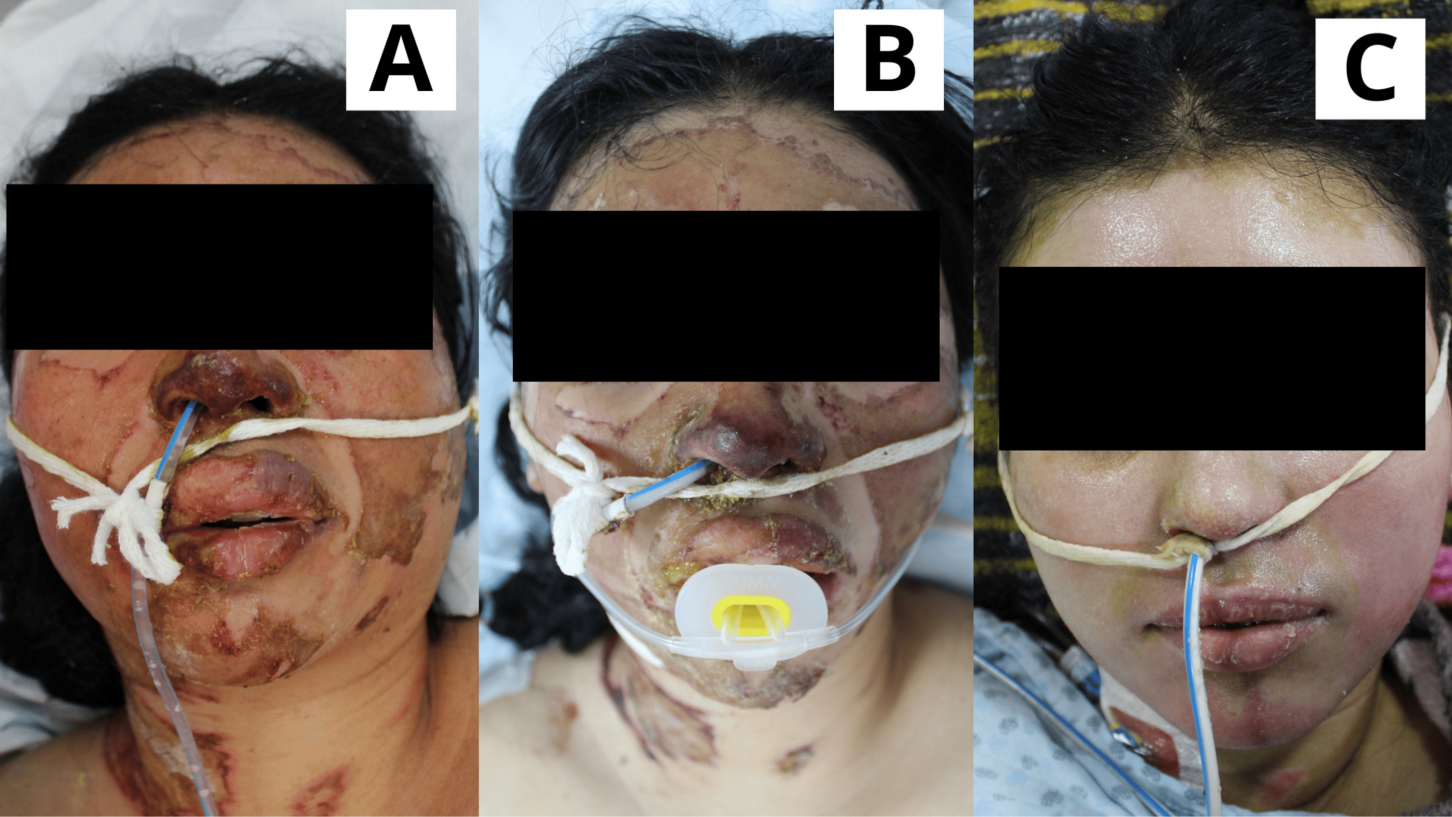
Case 7 Sixteen-year-old female patient with a diagnosis of a direct flame burn affecting the facial region. A: Day 1: Early burn excision was performed, and the Poloxamer 188 dressing was applied. B: Day 5: Lesions were progressing toward epithelialization, with the presence of eschar covering the wounds. C: Day 15: The face was completely epithelialized.

Among patients older than five years (5/7; 71.4%) who were able to verbalize their experience during treatment, all described the application of the dressing as “pleasant” or “refreshing” and reported a subjective reduction in pruritus during the healing process. The results of the present study are summarized (Table 1). Follow-up photographs are presented below (Figures 1-7).

## Discussion

Surfactants are surface-active compounds that can be classified into two main groups: ionic (anionic, cationic, and zwitterionic) and non-ionic types. Non-ionic surfactants do not ionize in water and contain hydrophilic groups, with poloxamers, also commercially known as Pluronics, falling into this category. The commercial nomenclature further specifies the physical state of the poloxamer, indicating whether it is a liquid (L), paste (P), or flake (F). The first number, when multiplied by 300, provides an estimate of the molecular weight of the polypropylene oxide (PPO) block, while the last number, multiplied by 10, indicates the percentage of polyethylene oxide (PEO) present in the poloxamer structure 8,9 Poloxamer 188 (P188), also known as Pluronic F68, is a triblock copolymer consisting of a hydrophobic PPO core flanked by two hydrophilic PEO chains at both ends [11-13].

Partial-thickness burns heal through re-epithelialization, as the dermis and its associated adnexal structures remain preserved. Several factors can enhance the speed of this process. Notably, epithelial migration occurs more rapidly in a moist wound environment than in dry conditions. When wounds are allowed to dry, exudate forms a crust that epithelial cells must enzymatically degrade before migration can occur, delaying wound closure and increasing the risk of hypertrophic scar formation. Topical agents that maintain wound moisture are therefore highly effective. Although certain biological dressings can adhere to the wound surface and sustain an optimal moist environment, their use on facial burns is limited due to difficulties in fixation and maintenance [14].

If facial burns fail to close within two to three weeks, skin grafting should be considered, taking into account the size of the defect and its relationship to facial aesthetic units. Scars within a single aesthetic unit are more conspicuous, whereas those placed at unit junctions are less noticeable; therefore, surgical planning should aim to position graft margins accordingly. Graft outcomes also depend on the recipient site: the forehead, with relatively immobile skin over a firm bony surface, generally provides better graft stability and less contraction, while grafts placed on the cheeks and lower face are more prone to contraction due to greater tissue mobility [4].

In burn treatment, hydrogels have emerged not merely as passive dressings but as active transdermal drug delivery systems. Owing to their three-dimensional network of cross-linked polymers with a high-water content, they create an ideal moist environment for wound healing and provide a physical barrier against external agents. However, the formation of eschar in deep burns represents a significant barrier to the passive diffusion of therapeutic agents, thereby limiting the effectiveness of hydrogels when used solely as dressings. Preclinical studies have demonstrated that the use of Poloxamer 188 as a delivery vehicle - for instance, in studies employing it to load proteins such as hFGF2 (human fibroblast growth factor 2) - enables controlled release, promotes cell proliferation and angiogenesis, and accelerates re-epithelialization in murine models of second-degree burns [14].

In a pediatric case series, the micellar matrix PluroGel demonstrated favorable outcomes in both superficial and deep partial-thickness thermal burns, as well as in other complex lesions, including infected pressure ulcers, friction injuries, calcium extravasation, and amniotic bands. In all patients, complete debridement was achieved without pain and without the need for mechanical methods, owing to its micellar mechanism, which softens and detaches devitalized tissue while retaining debris within its hydrophobic core. Its thermogel behavior also facilitated dressing changes, as lowering the temperature softens the gel, reducing adherence and pain, and allowing atraumatic application using a “no-touch” technique. Debridement was achieved within three to 10 days in thermal burns and approximately three weeks in infected ulcers, with a concomitant reduction in biofilm, viscous exudate, odor, and bacterial burden. Wound healing was rapid relative to severity: closure within seven to 14 days in superficial burns; 90% epithelialization by day 15 and complete closure by day 23 in deep burns without visible scarring; three weeks in calcium extravasation; 90% epithelialization by day eight in amniotic bands; and complete closure within 15 days in the infected pressure ulcer. Notably, most families were able to continue treatment at home without complications, thereby reducing hospitalizations and surgical interventions and promoting aesthetically and functionally favorable healing in anatomically sensitive regions such as the face, hands, and fingers [7].

Plurogel has demonstrated promise as a treatment for wounds infected by biofilms. In this study, we observed that Plurogel and its PSSD formulation (a surfactant-based dressing containing 1% silver sulfadiazine) had significant effects on the modulation of Staphylococcus aureus biofilms in vitro, reducing their formation and, in the case of PSSD, promoting complete biofilm eradication. The ability of Plurogel to prevent biofilm formation was observed when used as a wound pretreatment, by regulating the biological response to chronic infections. One of the most noteworthy findings was the reduction in inflammatory cytokine levels, particularly IL-6 and TNFα, highlighting its capacity to attenuate the chronic inflammation associated with bacterial biofilms. Another important aspect was cell viability; the data showed that Plurogel is not cytotoxic, thereby promoting cellular regeneration without causing additional damage to healthy tissue. Taken together, these results suggest that Plurogel, both in its standard formulation and when combined with PSSD, has substantial potential as a treatment for biofilm-infected wounds. Its ability to reduce inflammation, prevent biofilm formation, and promote cell viability makes it a viable option for the treatment of chronic wounds, such as those caused by persistent bacterial infections. However, although significant improvements were observed, the persistence of some biofilms in certain models suggests that further optimization of the formulation or application protocols may be required to achieve more consistent outcomes across all cases [15].

In a study comparing the cell viability and debridement efficacy of different antimicrobial wound dressings, the concentrated surfactant gel containing polyhexamethylene biguanide (CSG-PluroGel, CSG-PHMB) demonstrated lower cytotoxicity, maintaining higher viability of L929 and HDFa cells in assays when compared with dressings containing benzalkonium chloride (BXG) or polyhexamethylene biguanide alone (PXG). Specifically, CSG-PHMB promoted the removal of slough by loosening the collagen matrix and reducing surface tension, thereby facilitating the mobilization of wound fragments and debris. This effect was confirmed in vitro using a fluorescent artificial wound eschar (fAWE) model. These findings suggest that CSG-PHMB is a promising and less cytotoxic option for the treatment of chronic wounds, given its reduced impact on cell viability and effective debridement capability [16].

Other topical biomaterials have also been evaluated in burn care, such as Aloe vera-based hydrogels. In a recent systematic review and meta-analysis, four randomized controlled trials were included, comprising a total of 278 adult patients (18-65 years) with partial-thickness burns treated with Aloe vera gel. The results showed that its application significantly accelerated epithelialization compared with silver sulfadiazine, nitrofurazone, or placebo, achieving epithelialization within approximately two weeks, whereas control groups reached epithelialization at around three weeks. In addition, reductions in pain and pruritus at burn sites were observed, along with increased angiogenesis and fibroblast proliferation. These effects are attributed to antibacterial and anti-inflammatory properties, as well as stimulation of collagen, hyaluronic acid, and proteoglycan synthesis. Although the evidence is focused on adults, the trend toward the use of advanced topical dressings supports the notion that hydrogels can accelerate wound healing and reduce discomfort during recovery [17].

In a prospective, randomized, controlled study conducted in 168 patients with deep second-degree burns undergoing escharotomy, the use of hydrogel dressings covered with petrolatum gauze was compared with petrolatum gauze alone. Patients treated with hydrogel dressings experienced easier dressing removal, reduced adherence, and less pain during the first four dressing changes, as well as wounds that were moister, with less exudate and faster healing. This group showed significantly higher healing rates on postoperative days three, six, and 15, a lower proportion of positive bacterial cultures on days six and 15 and required fewer dressing changes. In addition, the need for skin grafting was substantially lower (14.3% vs. 40.5%), and the time to complete wound healing was markedly reduced (17.6 ± 2.8 days vs. 27.1 ± 3.0 days). At six months, patients in the hydrogel group exhibited less scar hyperplasia as assessed by the Vancouver Scar Scale. Overall, the use of hydrogel dressings following escharotomy was shown to improve local hydration, reduce pain and adherence, decrease infection rates and the number of dressing changes, accelerate healing, and reduce the need for skin grafting, although their higher cost may influence treatment selection depending on patient resources [18].

This case series reflects the clinical behavior of pediatric facial and scalp burns managed under a structured protocol combining early excision and standardized daily application of a Poloxamer 188-based dressing. The mean time to epithelialization observed in this cohort is consistent with expected healing trajectories for superficial and mixed partial-thickness burns treated with timely surgical debridement, suggesting that the incorporation of this dressing did not delay wound closure in anatomically sensitive areas. The longer epithelialization time observed in mixed-depth burns compared to purely superficial injuries supports the biological coherence of the findings and reinforces burn depth as the primary determinant of healing dynamics within the cohort.

Differences in healing patterns according to burn mechanism, particularly the variability seen in flame-related injuries, likely reflect differences in thermal exposure intensity and tissue penetration rather than a product-specific effect. Importantly, no infectious complications were documented despite exclusive reliance on this dressing during the acute phase, which suggests that its use was compatible with maintaining local wound stability in a region traditionally considered high risk due to sebaceous density and constant exposure.

This study should be interpreted within the context of its design as a retrospective case series. By nature, case series lack a control group and randomization, limiting the ability to establish comparative effectiveness or causal relationships. The small sample size and single-center experience further restrict external validity and generalizability. Additionally, although follow-up for scar prevention and rehabilitation is routinely conducted in our unit, a standardized protocol for long-term aesthetic and functional outcome assessment was not implemented, as the primary objective of this study was to evaluate the dressing during the acute epithelialization phase. Consequently, long-term scar quality, pigmentary alterations, and functional sequelae were not systematically measured, which represents an important limitation and an area requiring prospective evaluation in future studies.

Although the findings of this retrospective case series are limited by the small sample size, absence of a control group, and short-term follow-up, they provide relevant preliminary evidence supporting the safety and potential utility of Poloxamer 188-based dressings in pediatric burn care.

## Conclusions

The use of a Poloxamer 188-based dressing (PluroGel©) in pediatric patients with facial and scalp burns was associated with a favorable clinical course, characterized by good tolerability, atraumatic dressing changes, and timely epithelialization in anatomically sensitive regions. The physicochemical properties of this thermoresponsive micellar hydrogel, including its ability to maintain a moist wound environment, facilitate painless debridement, and minimize tissue adherence, support its clinical applicability in the conservative management of superficial and deep partial-thickness burns in children. Further prospective, controlled studies with larger cohorts and longer follow-up are warranted to confirm these observations and to better define their role in optimizing functional and aesthetic outcomes in pediatric facial and scalp burns.

## References

[1] Jeschke MG, van Baar ME, Choudhry MA, Chung KK, Gibran NS, Logsetty S (2020). Burn injury. Nat Rev Dis Primers.

[2] (2025). Burns. https://www.who.int/news-room/fact-sheets/detail/burns.

[3] Demir Yiğit Y, Yiğit E (2023). Pediatric craniofacial and neck burns. Indian J Otolaryngol Head Neck Surg.

[4] Greenhalgh DG (2020). Management of facial burns. Burns Trauma.

[5] Menon S, Jacques M, Harvey JG, Holland AJ (2015). Pediatric scalp burns: hair today, gone tomorrow?. J Burn Care Res.

[6] Percival SL, Mayer D, Malone M, Swanson T, Gibson D, Schultz G (2017). Surfactants and their role in wound cleansing and biofilm management. J Wound Care.

[7] Kirsner RS, Amaya R, Bass K (2019). Effects of a surfactant-based gel on acute and chronic paediatric wounds: a panel discussion and case series. J Wound Care.

[8] Rossani G, Hernández I, Alcolea JM, Castro-Sierra R, Pérez-Soto W, Trelles MA (2014). Tratamiento de quemaduras mediante plasma rico en plaquetas (PRP): parte I. Cir Plast Ibero-Latinoam.

[9] Murari A, Singh KN (2019). Lund and Browder chart-modified versus original: a comparative study. Acute Crit Care.

[10] Fontaine M, Ravat F, Latarjet J (2018). The e-burn application - a simple mobile tool to assess TBSA of burn wounds. Burns.

[11] Percival SL, Chen R, Mayer D, Salisbury AM (2018). Mode of action of poloxamer-based surfactants in wound care and efficacy on biofilms. Int Wound J.

[12] Zarrintaj P, Ramsey JD, Samadi A (2020). Poloxamer: a versatile tri-block copolymer for biomedical applications. Acta Biomater.

[13] Shang Y, Zhu S, Nie F (2022). Prospective application of poloxamer 188 in plastic surgery: a comprehensive review. Chin J Plast Reconstr Surg.

[14] Goh M, Du M, Peng WR, Saw PE, Chen Z (2024). Advancing burn wound treatment: exploring hydrogel as a transdermal drug delivery system. Drug Deliv.

[15] Salisbury AM, Mayer D, Chen R, Percival SL (2018). Efficacy of concentrated surfactant-based wound dressings in wound repair and biofilm reduction. Adv Wound Care (New Rochelle).

[16] Chen R, Salisbury AM, Percival SL (2020). A comparative study on the cellular viability and debridement efficiency of antimicrobial-based wound dressings. Int Wound J.

[17] Mahendra RE, Burhan A (2024). The effect of Aloe vera hydrogel on the process of burn healing: a systematic review and meta-analysis. J Wound Res Technol.

[18] Shang NS, Cui BH, Wang C, Gao H, Xu B, Zhao R, Huo R (2021). [A prospective randomized controlled study of the application effect of hydrogel dressings on deep partial-thickness burn wounds after dermabrasion and tangential excision]. Zhonghua Shao Shang Za Zhi.

